# Anordrin Eliminates Tamoxifen Side Effects without Changing Its Antitumor Activity

**DOI:** 10.1038/srep43940

**Published:** 2017-03-07

**Authors:** Wenwen Gu, Wenping Xu, Xiaoxi Sun, Bubing Zeng, Shuangjie Wang, Nian Dong, Xu Zhang, Chengshui Chen, Long Yang, Guowu Chen, Aijie Xin, Zhong Ni, Jian Wang, Jun Yang

**Affiliations:** 1 Key Laboratory of Contraception Regulation of National Population and Family Planning Commission, Shanghai Institute of Planned Parenthood Research, School of Pharmacy, Fudan University, 826 Zhangheng Road, Shanghai, 200032, China; 2 Shanghai Key Laboratory of Chemical Biology, School of Pharmacy, East-China University of Science, and Technology, 130 Meilong Road, Shanghai, 200237, China; 3 Obstetrics and Gynecology Hospital, Shanghai Ji Ai Genetics and IVF Institute, Institute of Reproduction and Development, Fudan University, Shanghai, 200011, China; 4 The First Affiliated Hospital, Department of Pulmonary Medicine, Chen, Wenzhou city, zhejiang Province, China; 5 New Drug Research and Development Center, School of Pharmacy, Second Military Medical University, Shanghai, China; 6 Institute of Life Sciences, Jiangsu University, Zhenjiang 212013, China

## Abstract

Tamoxifen is administered for estrogen receptor positive (ER^+^) breast cancers, but it can induce uterine endometrial cancer and non-alcoholic fatty liver disease (NAFLD). Importantly, ten years of tamoxifen treatment has greater protective effect against ER^+^ breast cancer than five years of such treatment. Tamoxifen was also approved by the FDA as a chemopreventive agent for those deemed at high risk for the development of breast cancer. The side effects are of substantial concern because of these extended methods of tamoxifen administration. In this study, we found that anordrin, marketed as an antifertility medicine in China, inhibited tamoxifen-induced endometrial epithelial cell mitosis and NAFLD in mouse uterus and liver as an anti-estrogenic and estrogenic agent, respectively. Additionally, compared with tamoxifen, anordiol, the active metabolite of anordrin, weakly bound to the ligand binding domain of ER-α. Anordrin did not regulate the classic estrogen nuclear pathway; thus, it did not affect the anti-tumor activity of tamoxifen in nude mice. Taken together, these data suggested that anordrin could eliminate the side effects of tamoxifen without affecting its anti-tumor activity.

Tamoxifen was the first FDA-approved drug for breast cancer patients with positively expressed estrogen receptors (ER)^1^. However, tamoxifen also induces side effects, such as uterine endometrial cancer and non-alcoholic fatty liver disease (NAFLD). Steatohepatitis may also develop, particularly in overweight women administered with tamoxifen. Importantly, for women with ER-positive (ER^+^) cancer, continuing tamoxifen treatment for up to 10 years, rather than stopping at 5 years, produces further reductions in recurrence and mortality, particularly after year 10^2^. Tamoxifen was also the first FDA-approved chemopreventive agent for those deemed at high risk for the development of breast cancer^3^. Because patients had to administer tamoxifen for more than 10 years, the side effects were a substantial concern. Previous studies into the molecular mechanism of tamoxifen-induced side effects resulted in the discovery of the classic estrogen nuclear pathway and membrane-initiated estrogen signal (MIES) pathways, which are modulated by membrane-bound estrogen receptors, orphan G-protein coupled estrogen receptor 30 (GPER1) and ER-α–36^4^,^5^,^6^. However, when investigators studied the physiological functions of *GPER1* and *ER-*α–*36*, they found that changing the expression of *GPER1* in cells also influences endogenous *ER-*α*-36* expression and *vice versa*^7^, suggesting that it remains unclear whether tamoxifen modulation of ER activity and its side effects are regulated by GPER1 and/or ER-α-36^8^,^9^. To eliminate tamoxifen-induced side effects and understand the physiological functions of GPER1 and ER-α-36, we screened selective estrogen receptor modulators, which, compared with tamoxifen, clinically exerted the opposite estrogenic activity in MIES. We then found that anordrin and tamoxifen may oppositely modulate MIES.

Anordrin/anordiol was synthesized using androgens. Interestingly, anordrin/anordiol specifically binds to ER rather than androgen or progestin receptors (AR or PR) and does not compete with ^3^H-labeled corticosterone for binding to serum proteins^10^. As a specific estrogen receptor antagonist on the uterus, anordrin has been marketed as an antifertility medicine under the brand name AF-53 in China since 1976. In this study, we found that anordrin and tamoxifen exerted opposing estrogenic effects to modulate the physiological function of MIES in a mouse uterus and liver. Combined administration of tamoxifen with anordrin can eliminate the side effects of tamoxifen without affecting its anti-tumor activity in nude mice.

## Results

### Anordrin inhibited tamoxifen-induced mitosis of endometrial epithelial cells in mouse uterus

**Figure 1:**
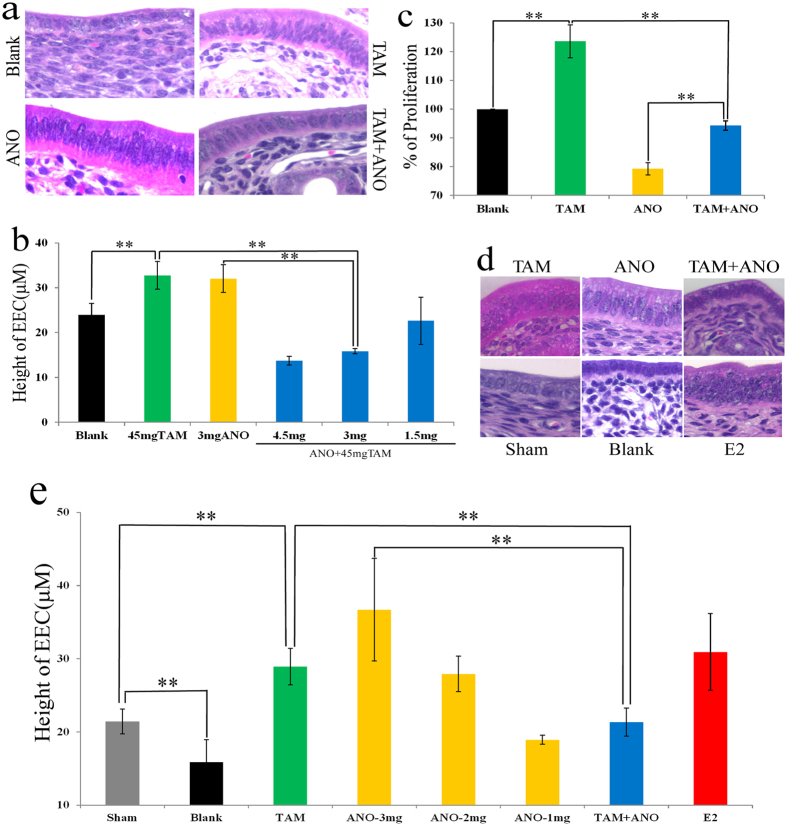
Anordrin inhibited tamoxifen-induced mitosis of EECs, as an anti-estrogenic agent in mice uterus. (**a**) Paraffin-embedded H&E sections (40x magnifications) of EECs from mice uterus treated by drugs for two weeks. TAM or ANO: mice were treated with tamoxifen (TAM) or anordrin (ANO) alone, respectively. TAM + ANO mice were treated with the combination of tamoxifen and anordrin. Blank mice were treated by vehicle. (**b**) Statistical analysis of the EEC height (μm), as measured from H&E-stained sections a. Blue bar: mice were administered with tamoxifen (TAM) + anordrin (ANO) (TAM + ANO) at the indicated doses. N = 2 × 6. **means P < 0.01. (**c**) Anordrin inhibited tamoxifen-induced Hec1A cell proliferation. TAM or ANO: Hec1A cells were treated with 4 μM tamoxifen (TAM) or 4 μM anordrin (ANO) alone, respectively. TAM + ANO: Hec1A cells were treated with 4 μM tamoxifen + 4 μM anordrin. Blank, Hec1A cells were treated by vehicle. N = 3 × 3; **means P < 0.01. (**d**) H&E staining of uterine EECs of OVX mice. (**e**) Statistical analysis of the EEC height (μm), as measured from H&E-stained sections d. Two independent experiments were performed with a total of twelve mice for each dose under the same conditions; **means P < 0.01.

**Figure 2:**
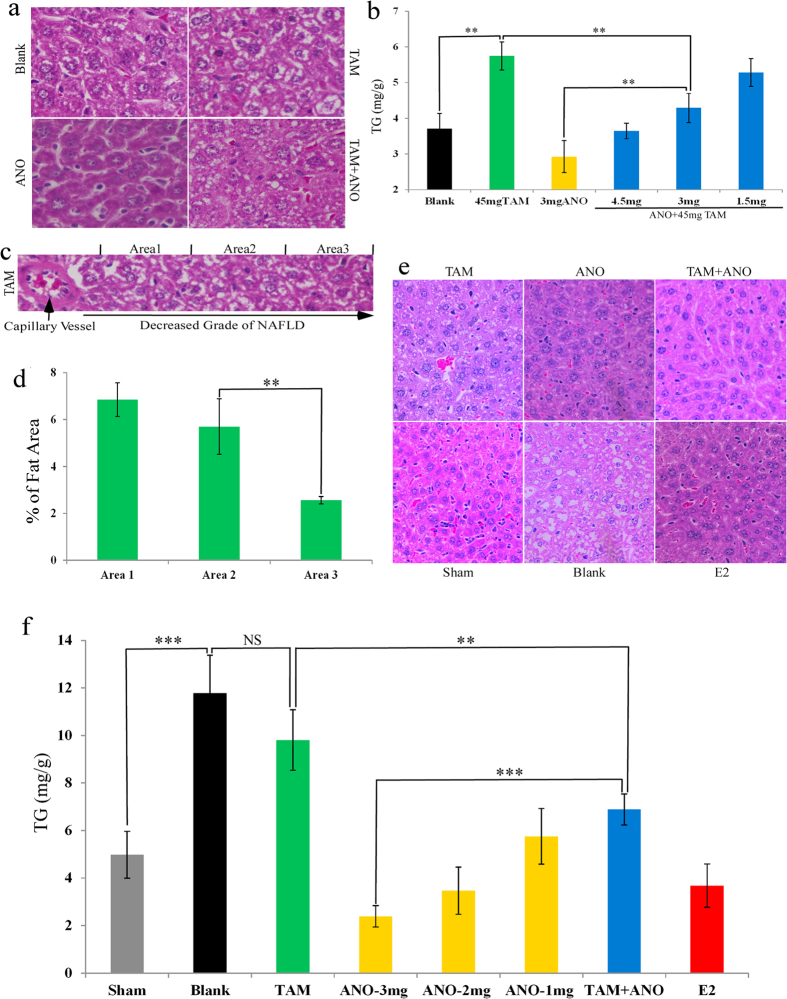
Anordrin inhibits tamoxifen-induced NAFLD as an estrogenic agent in mouse liver. (**a**) Paraffin-embedded H&E sections (40x magnifications) of normal mouse liver treated by drugs for 109 days. TAM or ANO: mice were treated with tamoxifen or anordrin alone, respectively. TAM + ANO mice were treated with the combination of tamoxifen and anordrin. Blank, no drug was added to the food. (**b**) Statistical analysis of liver triglyceride (TG) content, as in (**a**); N = 12; **means P < 0.01. (**c**) The worst NAFLD syndrome is shown close to the capillary vessel in the mouse liver. (**d**) Statistical analyzing the square of Fatty area as in (**c**); N = 2 × 6; **means P < 0.01. (**e**) H&E staining liver sections of OVX mice. (**f**) Statistical analysis of liver triglyceride (TG) content, as in (**e**). **and ***mean P < 0.01 and P < 0.001: Two independent experiments with total twelve sections, for each time point, under the same conditions.

Because tamoxifen induces the mitosis of endometrial epithelial cells (EEC), as an estrogenic agent^4^,^5^,^6^,^11^, it is considered the mechanism of tamoxifen-induced uterine endometrial cancer. Estrogen-induced mitosis of EEC is required for embryo implantation^11^. Anordrin inhibits embryo implantation to elicit its contraceptive effects^12^. These clinical indications suggest that anordrin and tamoxifen might modulate the proliferation of EEC in the opposite manner. We fed normal mice with drugs in food. The amount of tamoxifen and anordrin were determined dependent on their clinical dosages and the average amount of daily food uptake by the mice. After two weeks, the mice were sacrificed and their uteri were harvested. Twelve mice uterus of each group were fixed by paraformaldehyde. Hematoxylin and eosin (H&E) staining of paraffin-embedded uterine sections revealed that 3 mg anordrin (ANO) per kilogram food (3 mg/kg), and 45 mg tamoxifen (TAM) per kilogram food (45 mg/kg) increased the thickness of the EEC (Fig. 1a and b, TAM (green bar), ANO (yellow bar)); however, mitotic EEC did not occur in the anordrin (ANO) group (Fig. 1a, ANO). This result suggests that anordrin caused EEC hypertrophy rather than mitosis. In the tamoxifen + anordrin (TAM + ANO) group, mice were treated with 45 mg tamoxifen (TAM) + 1.5–4.5 mg anordrin (ANO) per kilogram food. At the 3-mg dosage of anordrin, the thickness of the EEC remained similar to that in the control group (Blank) (Fig. 1a and b, TAM + ANO (blue bar) vs. Blank (black bar)). These results indicated that anordrin inhibited tamoxifen-induced EEC mitosis, tamoxifen inhibited anordrin-induced EEC and uterine hypertrophy, and the combined administration of tamoxifen with anordrin restored the EEC to normal, suggesting that anordrin and tamoxifen exerted the opposite effect in the EEC of a mouse uterus. In addition of EEC phenotype, anordrin and tamoxifen also exerted the opposite effect in the modulation of mouse uterine hypertrophy (SI. Fig. 2).

Hec1A is a human uterine endometrial cancer cell (hECC), which only expresses ER-α-36 and not ER-α-66^13^. Tamoxifen stimulated Hec1A cell proliferation via MIES, as an ER-α-36 agonist^13^. To verify whether anordrin could inhibit tamoxifen-stimulated proliferation of endometrial cancer cells, Hec1A cells were treated with tamoxifen (TAM) or anordrin (ANO) or tamoxifen + anordrin (TAM + ANO) for 6 days *in vitro*, and then we counted the number of living Hec1A cells and normalized to Blank (vehicle) (Fig. 1c). The result confirmed that tamoxifen (TAM) stimulated Hec1A proliferation (Fig. 1c, TAM (green bar) vs blank (black bar)). Anordrin (ANO) exerted the opposite effect to inhibit Hec1A proliferation stimulated by tamoxifen (TAM) (Fig. 1c, TAM + ANO (blue bar) vs. TAM (green bar)), suggesting that anordrin may be an ER-α-36 antagonist to inhibit tamoxifen-induced endometrial cancer cell proliferation *in vitro*.

**Figure 3:**
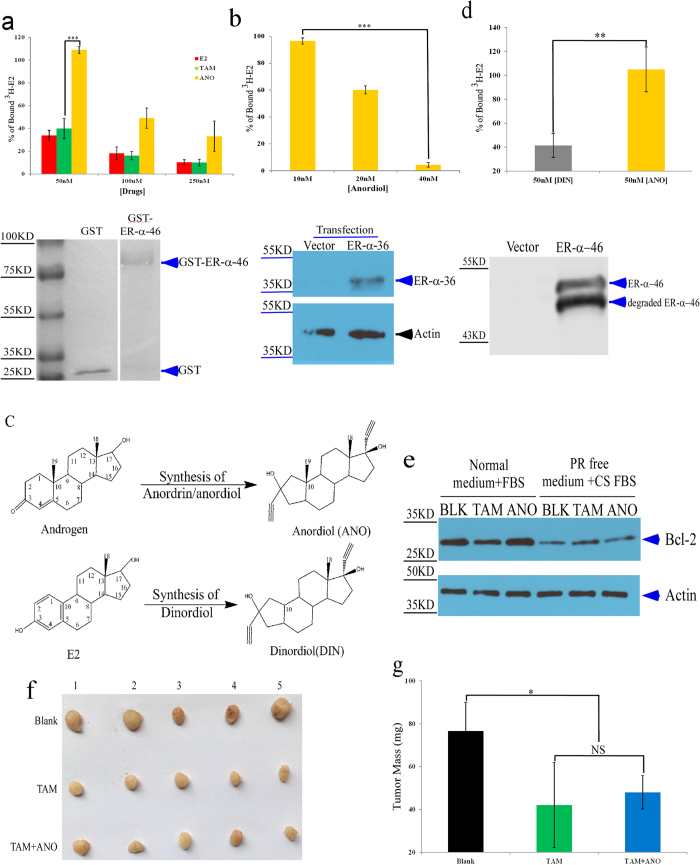
Anordrin (ANO) does not affect the anti-tumor activity of tamoxifen. (**a**) Upper panel: The percent of ^3^H-E2 binds to GST-ER-α-46 competed by E2 or TAM (tamoxifen) or ANO (anordiol) to be normalized with ^3^H-E2 only (blank), after subtracting the DPM of3 H-E2 from equal molar amount of GST protein on beads N = 3 × 3; ***means P < 0.001; Lower panel: SDS-PAGE followed by Coomassie Blue R250 staining to show the GST and GST-ER-α-46 fusion protein after purification using glutathione beads. (**b**) Upper panel: The percent of ^3^H-E2 binds to ER-α-36 competed by ANO (anordiol) to be normalized with ^3^H-E2 only (blank), after subtracting the DPM of ^3^H-E2 from an equal amount of total cellular protein; N = 3 × 3; ***means P < 0.001; Lower panel: SDS-PAGE followed by western blotting to show the ER-α-36 expression by vector and ER-α-36 plasmid after 24 hours of transient transfection. (**c**) The molecular structure of androgen vs. E2 and Anordiol (ANO) vs. Dinordiol (DIN). (**d**) Upper panel: The percent of ^3^H-E2 binds to ER-α-46 competed by anordiol (ANO) and dinordiol (DIN) to be normalized with ^3^H-E2 only (blank), after subtracting the DPM of 3H-E2 from an equal amount of total bacterial protein. N = 3 × 3; **means P < 0.01; Lower panel: SDS-PAGE followed by western blotting to show ER-α-46 expression in crude *E. coli* lysate. (**e**) The expression of Bcl-2 protein in MCF-7 cells, detected by western blotting, is regulated by estrogen and tamoxifen after 48 h. The upper gel shows the expression of Bcl-2 in MCF-7 cells treated with 7.5 μM tamoxifen (TAM) or 7.5 μM anordrin (ANO), or DMSO (blank, BLK), as assessed by western blotting using anti-Bcl-2 antibodies (PR free: phonel red free; CS: charcoal stripped; FBS: fetal bovine serum). The lower gel shows western blotting for actin to confirm equal protein loading. (**f**) The xenograft of MCF-7 cells in nude mice treated by vehicle (Blank), tamoxifen (TAM) and tamoxifen + anordrin (TAM + ANO). (**g**) Statistical analysis of the MCF-7 xenograft mass from nude mice, as in (**f**). Two independent experiments were performed. More than nine tumor mass was measured for statistical analysis in each group under the same conditions. *means P < 0.05.

To assess the anordrin inhibition of tamoxifen-induced EEC mitosis, as an antiestrogenic effect in a mouse uterus, ovariectomized mice (OVX) were treated with anordrin (3 mg/kg) and tamoxifen (45 mg/kg) via food uptake for 2 weeks. Twelve mice uterus of each group were fixed by paraformaldehyde. H&E sections of OVX mice uteri showed that anordrin (ANO) and tamoxifen (TAM) induced hypertrophy and mitosis of EEC, as estrogenic agents, respectively (Fig. 1d, ANO, TAM). Increased thickness of the EEC was exhibited in both the anordrin (ANO) and tamoxifen (TAM) groups (Fig. 1d and e, ANO (yellow bar), TAM (green bar)). Importantly, the thickness of the EEC in the 45 mg tamoxifen + 3 mg anordrin group (TAM + ANO) was decreased to the comparable height as in the sham group (Fig. 1e, TAM + ANO (blue bar) vs. TAM (green bar) or ANO (yellow bar)). The phenotype of the EEC H&E sections in the tamoxifen + anordrin (TAM + ANO) group was similar to the sham group (Fig. 1d, TAM + ANO, sham). These data suggested that anordrin and tamoxifen exerted the opposite estrogenic effect on the EEC of a mouse uterus. In addition, anordrin prevented uterine atrophy in OVX mice (SI1 Fig. 3). Tamoxifen inhibited anordrin prevention of uterine atrophy under the same testing condition (SI1 Fig. 3), suggesting that anordrin and tamoxifen exerted the opposite estrogenic effect on the mouse uterus, and further reinforced that anordrin and tamoxifen may exert the opposite estrogenic effect on the EEC of mouse uterus.

### Anordrin inhibits tamoxifen-induced NAFLD in mice liver

Tamoxifen increases the hepatic fat content by inhibiting the role of estrogen in maintaining hepatic lipid homeostasis^14^. Anordrin induced hypertrophic phenotype in mouse EEC, implying that anordrin may up-regulate metabolism as an estrogenic agent (Fig. 1d, ANO). Tamoxifen inhibited anordrin-induced EEC hypertrophy (Fig. 1d, TAM + ANO vs. ANO), suggesting that tamoxifen and anordrin modulate metabolic effect of estrogen oppositely. We then hypothesized that anordrin can inhibit tamoxifen-induced NAFLD in a mouse liver. Female mice that were 7–8 weeks old were fed with drugs in food for 109 days. At least twelve livers were harvested from the experimental groups. Twelve pieces of mice livers from each group were fixed by paraformaldehyde, and paraffin sections were prepared for H&E staining. Additionally, 30–50-mg pieces of liver tissue were incubated in lipid extraction solution and 0.5 ml physiological salt solution. The amount of total cholesterol (TC) and triglycerides (TG) in the organic phase was then measured using TC and TG assay kits, respectively. The analysis of H&E-staining liver sections revealed that compared with Blank (no drug in food), there was an increase of NAFLD syndrome in the tamoxifen (TAM) group (TAM; fed by 45 mg tamoxifen per kilogram food (45 mg/kg)), (Fig. 2a and b, TAM (green bar) vs. Blank (black bar)). The NAFLD grade was significantly increased in liver cells that were close to the capillary vessels (Fig. 2c and d). Additionally, compared with blank, hepatic TG levels were also increased in the tamoxifen (TAM) group, (Fig. 2b, TAM (green bar) vs. Blank (black bar)). The opposite effect was revealed in the anordrin group (ANO fed 3 mg anordrin per kilogram food (3 mg/kg)) (Fig. 2a and b, ANO (yellow bar)). Importantly, anordrin can reverse tamoxifen-induced NAFLD syndrome and decrease liver TG content induced by 45 mg/kg tamoxifen in a dose-dependent manner (Fig. 2b, TAM + ANO group (blue bar) vs TAM (green bar) or ANO (yellow bar)). There were no significant differences in food uptake and hepatic TC levels among groups (SI1 Fig. 4). These data suggested that anordrin could inhibit tamoxifen-induced NAFLD and liver TG phenotype *in vivo*.

To assess the anordrin modulation of liver TG content as an estrogenic effect, we decreased estrogen production using OVX mice. Mice were fed with drugs in food for 2 months. Following the same method as Fig. 2a, we observed that anordrin as well as E2 prevented TG content in the liver in a dose-dependent manner in OVX mice (Fig. 2e and f, ANO (yellow bar)). Compared with the tamoxifen (TAM) or anordrin (ANO) group, the liver TG content was significantly reversed in the tamoxifen + anordrin group (TAM + ANO) (Fig. 2f, TAM + ANO (blue bar) vs. TAM (green bar) or ANO (yellow bar)). These data suggested that anordrin could enhance liver TG metabolism as an estrogenic agent.

### Anordrin does not affect tamoxifen activity of ER^+^ breast cancer resistance in nude nice

Previously, Mehta *et al*. identified two estrogen-binding complexes, named 4 S and 8 S, according to their rate of ultracentrifugation sedimentation in the uterine cytosol of mice. Anordiol, the unesterified and active metabolite of anordrin, binds preferentially to the 8 S complex. In contrast, tamoxifen binds to both the 4 S and 8 S estrogen-binding complexes^10^. Importantly, estrogen regulates biological functions through two methods: the classic estrogen nuclear pathway and membrane-initiated estrogen signal pathways (MIES)^4^,^5^,^6^. The preferential binding of anordrin to one estrogen-binding uterine cytosolic complex suggests that anordin might only modulate one estrogenic pathway. Tamoxifen and estrogen may modulate both the classic estrogen nuclear pathway and MIES. We then assessed whether anordrin played a role in the classic nuclear pathway of estrogen modulation. Because the ER-β selective agonist was not as well suited as E2 and as an ER-α selective agonist to mediate uterine endometrial proliferation^15^, we only tested the binding affinity of anordiol to ER-α. ER-α-46 is an effective ligand-regulated transcription isoform of ER-α^16^,^17^. ER-α-46 was expressed in *E. coli* and purified using glutathione beads. The binding affinity of anordiol, tamoxifen, and E2 for the ER-α-46 fusion protein was then compared using a ^3^H-E2-competition assay. The results demonstrated that 50 nM anordiol could not inhibit the binding of 0.5 nM ^3^H-E2 to 1 μg ER-α-46; however, the same concentration of either tamoxifen or E2 blocked >60% of the binding between ^3^H-E2 and ER-α-46 (Fig. 3a, ANO (yellow bar) vs. TAM (green bar) or E2 (red bar)). However, 40 nM anordiol (ANO) as well as E2 and tamoxifen including its active metabolites (4hydroxytamoxifen (4HTAM) and endoxifen (END)) inhibits the binding of 0.5 nM ^3^H-E2 to ER-α-36 transiently expressed in HEK293 cells (Fig. 3b, SI1 Fig. 5), suggesting that anordiol binds preferentially to ER-α-36 as well as 8 S complex of uterine cytosol and then modulates MIES.

Because androgen contains a methyl group (C19) at the C10 site, instead of hydrogen at the same site of estrogen (E2) (Fig. 3c), androgen binds to the androgen receptor (AR) but does not bind to ER. Androgens are used to synthesize anordrin/anordiol. Interestingly, anordrin/anordiol specifically binds to ER rather than androgen or progestin receptors (AR or PR) and does not compete with ^3^H-labeled corticosterone for binding to serum proteins^10^. These data suggest that methyl group (C19) at the C10 site of anordrin is a crucial group, blocking anordiol-binding to the ligand binding domain (LBD) of ER. We then synthesized dinordiol (DIN) using estrogen (Fig. 3c). The binding affinity of anordiol (ANO) and dinordiol (DIN) with ER-α-46 indicated that the C19 group of anordiol (ANO) is crucial for the binding of anordiol to ER-α LBD (Fig. 3d, ANO (yellow bar) vs. DIN (brown bar)), suggesting that anordiol may not bind to ER-α in cytosol and does not regulate the classic nuclear estrogen pathway.

It is known that Bcl-2 is a key member of the anti-apoptotic family proteins. Its overexpression is linked to many kinds of cancers in humans. The *bcl2* promoter contains the estrogen response element (ERE) sequence. Therefore, the expression level of *bcl2 *mRNA in MCF-7 cells is regulated positively by E2 and inhibited by tamoxifen through the classic estrogen nuclear pathway^18^. In this study, our findings showed that the expression of Bcl-2 protein was enhanced by culture medium containing estrogen compared with that containing charcoal-stripped (CS) Fetal bovine serum (FBS) (Fig. 3e, BLK (Blank]). Furthermore, treatment with 7.5 μM tamoxifen (TAM) inhibited Bcl-2 expression (Normal FBS, Fig. 3e; TAM vs. BLK), whereas Bcl-2 expression was unaffected by treatment with 7.5 μM anordrin (ANO) (Normal FBS, Fig. 3e; ANO vs. BLK). Importantly, neither 7.5 μM tamoxifen (TAM) nor 7.5 μM anordrin (ANO) affected Bcl-2 expression in cells cultured in medium without estrogen (CS FBS in phenol red free (PR-free) EMEM medium; Fig. 3e, TAM; ANO). These results further reinforced our conclusion that anordrin was not involved in the classic nuclear pathway of estrogen regulation and suggested that anordrin may not affect tamoxifen activity to inhibit the growth of ER^+^ breast cancer.

Anordrin does not modulate the expression of cancer gene *via* the classic estrogen nuclear pathway and will not affect the activity of tamoxifen to inhibit the growth of ER^+^ breast cancer. We next tested the anti-tumor activity of tamoxifen containing anordrin *in vivo*. ER^+^ breast cancer cells, MCF-7, were used as a xenograft model in female nude mice^19^. A suspension of 1 × 10^7^ cells in 100 μL 1xDPBS with matrixgel was injected S.C. into mice. Tumors were grown until average tumor volume reached 0.1–0.2 cm^3^. Tamoxifen doses were determined dependent of the ref. 19, and the results are shown in Figs 1 and 2. Nude mice were then administered daily via intragastric injection for 3 weeks with a vehicle or 6 mg tamoxifen (TAM) per kilogram body mass, or the same amount of tamoxifen (TAM) + 0.4 mg anordrin (ANO). Compared with the vehicle (Blank), tamoxifen (TAM) significantly inhibited MCF-7 xenograft (Fig. 3f and g, Tam (green bar) vs Blank (black bar)). MCF-7 xenograft in the tamoxifen + anordrin (TAM + ANO) group exhibited similar mass to those mice in the TAM group (Fig. 3g, TAM (green bar) vs. TAM + ANO (blue bar)), suggesting that anordrin did not affect the tamoxifen activity of ER^+^ breast cancer resistance *in vivo*.

## Discussion

It is known that embryo implantation requires E2-induced EEC proliferation *via* ERs. The ER-β selective agonist and G1, a GPER1 selective agonist, were not as well suited as the E2 and ER-α selective agonist to mediate estrogenic effect in mice uterus^15^,^20^. Neither tamoxifen nor E2 induced EEC proliferation in ER-αKO mice^11^. *GPER1*KO mice revealed comparable fertility to wild-type mice^20^, suggesting that the E2 regulation of EEC proliferation requires ER-α but not ER-β and GPER1.

Anordrin was marketed as a contraceptive medicine because it is a specific ER antagonist on uterus^9^,^12^, indicating that anordrin is an ER-α antagonist. Three ER-α variants, ER-α–66, -46 and -36, were reported to regulate classic estrogen nuclear pathway and MIES^5^,^6^,^7^,^17^,^21^. ER-α–66 and -46 variants are an effective ligand-regulated transcription isoforms of ER-α^16^,^17^, which regulate RNA transcription *via* classic estrogen nuclear pathway^9^. The *bcl2* promoter contains the ERE sequence. The expression level of *bcl2* mRNA in MCF-7 cells is regulated positively by E2 and inhibited by tamoxifen through the classic estrogen nuclear pathway^18^. The current results demonstrated that anordrin bound to ER-α46 weakly compared with E2 and tamoxifen, and that E2 enhanced Bcl-2 expression; tamoxifem inhibited Bcl-2 expression; anordrin did not regulate Bcl-2 expression under the same condition in MCF-7 cells. These results indicated that tamoxifen is an antagonist of the classic estrogen nuclear pathway to exert its anti-ER-α positive tumor effect, and that anordrin did not regulate the classic estrogen nuclear pathway. Therefore, anordrin did not affect the antitumor activity of tamoxifen.

ER-α–36 is devoid of both the AF-1 and AF-2 transactivation domains of ER-α–66 and the E2-modulated activity of intrinsic RNA transcription^8^,^21^; it is found as predominantly a plasma membrane-associated ER-α(mER–α)variant to transduce E2-mediated MIES^5^,^6^,^7^,^8^,^21^. Hec1A is a human uterine endometrial cancer cells (hECC), which positively expressed ER-α–36 but not ER-α–66^13^. Tamoxifen stimulated Hec1A proliferation as an ER-α–36 agonist^13^; it clinically induced human endometrial cancer and mice EEC mitosis as estrogenic agent^11^; and that tamoxifen as well as E2 induced EEC mitosis in a manner that was dependent on ER-α but independent of the classic ER-α nuclear pathway^11^. These data suggested that tamoxifen is an agonist of mER-α and/or ER-α-36. The current results demonstrated that anordrin inhibited tamoxifen-induced EEC mitosis and hECC proliferation, suggesting that anordrin functions as an antagonist of mER-α and/or ER-α-36 to exert its anti-estrogenic and antifertility effects in uterus. Because our data showed that tamoxifen and anordrin exerted the opposite effects in the uterine EEC mitosis and human ECC proliferation, anordrin can eliminated tamoxifen-induced uterine endometrial cancer.

In addition, current data revealed that anordrin stimulated the hypertrophic phenotype in mice uterus EEC and inhibited TG accumulation in the liver of OVX mice as an estrogenic agent, suggesting that anordrin is an estrogenic agent of MIES to modulate TG metabolite in liver. The previous study suggested that tamoxifen increases the hepatic fat content by inhibiting the role of estrogen in maintaining hepatic lipid homeostasis^14^. Current data revealed that anordrin inhibited tamoxifen-induced TG accumulation in a mouse liver, suggesting that anordrin can eliminate tamoxifen-induced NAFLD. Taken all together, we conclude that anordrin can eliminate the side effects of tamoxifen without affecting its activity of breast cancer resistance. This conclusion will be further confirmed in clinical trial study.

## Methods

### Materials

E2 and androgen were purchased from the Okahata Trading Co., Ltd. (Shanghai, China). The detailed method for anordrin and dinordil synthesis was previously reported [Journal of Sichuan University (Medical Science Edition), 1976, 109–114]. All compounds were confirmed using ^1^H and ^13^C NMR spectra. Jian Wang generously provided the anordrin standard. The purity of the synthesized anordrin was verified using Agilent 1100 HPLC with an Anthane C18 column. Briefly, anordrin was solved in 100% methanol at a concentration of 0.1 mg/l, and 10 μl of anordrin solution was loaded onto the column at 30 °C. Anordrin was then eluted at ~88% [acetonitrile] using a graded elution program with a water/acetonitrile mixture (1:1) and 100% acetonitrile with a flow rate of 1 ml/min. Anordrin that was 98% pure was detected at an absorption wavelength of 208 nm.

Fetal bovine serum (FBS) was purchased from GIBCO (10099141; Made in Australia). E2, tamoxifen, and isoflavone (MPG USP grade) were obtained from Okahata Trading Co., Ltd. (Shanghai, China). All compounds were solved in DMSO before use. The mouse chow, AIN-93G, was purchased from Trophic Animal Feed High-tech Co., Ltd, Nantong, China. H&E sections were obtained from BK Animal Model, Inc. (Shanghai China). Chemicals including 4-hydroxytamoxifen and endoxifen were purchased from Sigma or Aladdin, unless otherwise stated. The TG (Cat^#^E1013) and TC (Cat^#^E1015) assay kits were purchased from Apply Gen Technologies, Inc. and Nanjing Jiancheng Bioengineering Institute, respectively. Bcl-2 (Cat^#^51-1513GR) antibody was purchased from BD Biosciences (USA). ER-α antibody (Cat^#^8644) and actin (Cat^#^4970 s) were purchased from Cell Signaling Technology (Danvers, MA USA). ^3^H-E2 and restriction enzymes were purchased from PerkinElmer and Takara Bio Inc, respectively.

### Plasmid construction, and protein expression, purification, and characterization using LC-MS/MS

ER-α-46 and ER-α-36 were cloned into pET-28 and pGEX-6P-1 using *Eco*RI and *Xho*I sites. Production of the His- or GST-ER fusion proteins was induced using 0.1 μM IPTG, and the proteins were expressed in *E. coli* at 25 °C for 3 hours. Bacteria were then harvested, and the GST fusion proteins were purified according to the manufacturer’s instructions (GE). The purified proteins were eluted from GSH-beads using 1 × sample buffer at 100 °C for 5 min, and the supernatants were separated on 10% gels using SDS-PAGE. The gels were stained with Coomassie blue-R250, and the amount of GST-ER-α-46 was determined using NIH Image J software using GST as the standard. Subsequently, the bands corresponding to GST-ER-α-46 were cleaved from the gel and characterized using LC-MS/MS. Human ER-α cDNAs were purchased from YR gene (China, Changsha) and subcloned into pEGFP-N1 vectors (Clontech). The PCR primers were designed from the human ER-α-36 cDNA sequence in NCBI.

### Cell culture, transfection, the induction of an MCF-7 stable cell line, and Hec1A cell proliferation assay

MCF-7, Hec1A and HEK-293 cells were grown according to the protocols recommended by ATCC. Cell transfection was performed using Lipofectamine 2000 (Invitrogen) according to the manufacturer’s instructions. The expression of GFP fusion protein was confirmed using western blotting. The proliferation assay of Hec1A cells were performed by counting the number of living Hec1A cell after inoculation for 6 days. Briefly, 5 × 10^3^ Hec1A cells containing drugs in 0.5 ml medium were inoculated into 24 well plates. Medium was exchanged once at the third day from initiation day. In the sixth day, Hec1A cells were trypsined up and stained by trypan blue (Cat#T8154, Sigma). Total living cells were counted using cell counter.

### The preparation of rabbit polyclonal anti-ER-α-36 antibody

The rabbit polyclonal anti-ER-α-36 antibody was prepared by custom service from GL Biochem Ltd. (Shanghai, China). Exactly following ref. 21, the C-terminal peptide of ER-α-36 was synthesized and coupled to hemocyanin as an antigen, and then anti-ER-α-36 antibody was raised.

### Drug-ER-α binding affinity and ^3^H-E2-drug competition assays

Assays were performed as refs 7 and 13 with brief modification. Purified GST-ER-α-46 was coupled on glutathione beads in TE buffer (50 mM Tris [pH 8.0], 10 mM EDTA, 20 mM β-Mercaptoethanol. Drugs and ^3^H-E2 were added to 1 ml protein solution, and incubated at 4 °C for 2 hours. Beads were washed twice using TE buffer and transferred to scintillation vials. The amount of bound ^3^H-E2 was then measured using a GM meter.

ER-α36 was transiently expressed in HEK293 cells using ER-α36 cDNA plasmid. After 24 h of transfection, cells were suspended in TE-glycerol buffer (10% glycerol, 50 mM Tris [pH 8.0], 10 mM EDTA, 20 mM β-Mercaptoethanol. After centrifugation at 500 g for 3 min, drugs and ^3^H-E2 were added to 1 ml supernatant (5 mg/ml), and incubated at 4 °C for 2 h. Supernatant was centrifuged at 30 krpm for 90 min. The pellet was transferred to scintillation vial to count the amount of bound ^3^H-E2.

His tagged ER-α–46 was expressed in *E. coli* and lysed in 1xPBS containing 10 mM EDTA, 20 mM β-Mercaptoethanol. Then, 1 ml crude lysate containing 10 mg total protein was mixed with drugs and ^3^H-E2 for 2 h. Next, 30 mg Norit A [Immunometrics LTD, London], suspended in 100 μl 0.3% Dextran-0.1 M sodium phosphate solution [Immunometrics LTD, London], was added into the reaction solution on ice for 15 min. After centrifugation at 4 °C for 12 min, 500 μl supernatant was transferred to scintillation vials to count the amount of bound ^3^H-E2. The bound ^3^H-E2 was calculated, after subtracting the DPM of ^3^H-E2 from an equal amount of total protein which bacterial was transformation using vector.

### Extraction of cell lysates, measuring the total protein concentration, and western blotting

Cells were harvested and lysed in RIPA buffer or 1 × DPBS containing 1% Triton X-100 and protease inhibitor cocktail (Sigma). The total protein concentration was then measured using a Bio-Rad protein staining dye. Western blotting was performed according to standard protocols using nitrocellulose membranes (Millipore) with Bio-Rad semidry transfer.

### Animals

ICR and nude mice strains were purchased from the Shanghai BK Animal Model Inc. Ltd., China. The animal experimental protocol was approved by Animal Ethics Committee of the Shanghai Institute of Planned Parenthood Research (SIPPR Regulation^#^2015–13), in accordance with the 588^th^ regulation of animal experiments issued by Chinese Government in 2011. All animal experiments were performed under audit of the SIPPR Animal Ethics Committee.

### Construction of the OVX mice model and drug administration

The ovaries of 7- to 8-week-old ICR mice (BK Animal Model, Inc. Shanghai) were excised surgically. After 3 days or 2 months, the drugs were administered daily using gastric tract injections or mixed with food and fed to mice. The dosage of 45 mg tamoxifen per kilogram food, or 0.5, 1, 1.5, 2, 3, 4.5, 9 mg anordrin per kilogram food, or mixture of the two drugs was administered to mice. The amount of drugs in food was designed dependent of the uptake of food by mice daily. The daily drug uptake in food is similar to the drug uptake with daily gastric tract injections. The mice were fed with AIN-93G (Blank) or AIN-93G mixed with drugs for 1, 2, 3, 4, 6, 9, 12, and 15 weeks and then sacrificed. The tissues were either frozen at −80 °C or paraffin-embedded for sectioning.

### Generating xenograft MCF-7 tumor in female nude mice

Five-week-old mice were administered twice per week with 1 mg E2 per kg body mass. After one week E2 administration, a suspension of 1 × 10^7^ MCF-7 cells in 100 μL 1xDPBS with 100 μl matrixgel was injected S.C. into nude female mice. Tumors were grown until average tumor volume reached 0.1–0.2 cm^3^. Nude mice were administered daily for 3 weeks with vehicle or 6 mg tamoxifen per kilogram body mass, or the same amount of tamoxifen plus 0.4 mg anordrin. Mice were sacrificed. Tumor mass was measured.

### Preparation and analysis of paraffin-embedded sections and H&E staining

Mouse tissues were harvested and fixed using 4% formaldehyde in 1 × DPBS (Solar Bioscience & Technology, Beijing, China). Paraffin sections were prepared and H&E staining was then performed according to the standard protocols at the GLP laboratory of BK Animal Model, Inc. The paraffin was removed from liver sections by washing with xylene three times, and the xylene was then removed using 100%, 95%, and 75% alcohol, sequentially. For antigen preparation, the sections were incubated with 0.01 M sodium citrate buffer (pH 6.0) at 95 °C for 2 min and washed using 1 × PBS three times for 5 min each. The sections were then incubated in 1 × PBS containing 3% H_2_O_2_ for 5–10 min at room temperature and washed using 1 × PBS for 5 min. The fatty area of liver H&E sections was assayed using NIH image J software.

### Measuring TC and TG in the liver and serum of mice

Mouse liver samples (30–50 mg) were harvested and homogenized in 1 ml of a chloroform:methanol (2:1) mixture and then extracted using 0.5 ml ddH_2_O. The organic phase was transferred to new tubes and left to dry in the air, and the TC and TG content was measured using a kit, according to the manufacturer’s instructions. The error was eliminated using an internal standard control.

### Statistical analysis

At least two repeats were performed for each drug dosage include vehicle (Blank) in all experiments. More than nine mice tissues or tumors were used for statistical analysis in all animal experiments. All data are presented as the mean ± SD. Asterisks indicate significant differences, as calculated using two-tailed Student’s *t*-test. A value of P < 0.05 was defined as significant. At least three repeats were used for all statistical analyses.

## Additional Information

**How to cite this article:** Gu, W. *et al*. Anordrin Eliminates Tamoxifen Side Effects without Changing Its Antitumor Activity. *Sci. Rep.* **7**, 43940; doi: 10.1038/srep43940 (2017).

**Publisher's note:** Springer Nature remains neutral with regard to jurisdictional claims in published maps and institutional affiliations.

## Supplementary Material

Supplementary Information
