# Supplementary material for: Anordrin Eliminates Tamoxifen Side Effects without Changing Its Antitumor Activity
Source: Sci Rep. 2017 Mar 7;7:43940. doi: 10.1038/srep43940 (PMC5339706; doi:10.1038/srep43940)

Anordrin Eliminates Tamoxifen Side Effects without Changing Its Antitumor Activity

Wenwen Gu[1#], Wenping Xu[2#], Xiaoxi Sun[3], Bubing Zeng[2], Shuangjie Wang[1], Nian Dong[4], Xu Zhang[1], Chengshui Chen[4], Long Yang[5], Guowu Chen[3], Aijie Xin[3], Zhong Ni[6], Jian Wang[1*], Jun Yang[1*]

1. Key Laboratory of Contraception Regulation of National Population and Family Planning Commission, Shanghai Institute of Planned Parenthood Research, School of Pharmacy, Fudan University, 826 Zhangheng Road, Shanghai, 200032 China

2. Shanghai Key Laboratory of Chemical Biology, School of Pharmacy, East-China University of Science and Technology, 130 Meilong Road, Shanghai, 200237 China

3. Obstetrics and Gynecology Hospital, Shanghai Ji Ai Genetics and IVF Institute, Institute of Reproduction and Development, Fudan University, Shanghai 200011, China

4. The First Affiliated Hospital, Department of Pulmonary Medicine, Chen, Wenzhou city, zhejiang Province, China

5. New Drug Research and Development Center, School of Pharmacy, Second Military Medical University, Shanghai, China

6. Institute of Life Sciences, Jiangsu University, Zhenjiang 212013, China

# Wenwen Gu and Wenping Xu contributed equally to this work.

*To whom correspondence is to be addressed: Shanghai Institute of Planned Parenthood Research, 2140 Xietu Road, Building 2, Room 502, Shanghai, People’s Republic of China, 200032. E-mail: junyangsd@yahoo.com or wangjiansippr@126.com

Supplementary Information (SI):

SI1. Figures:

SI2 fig. 1

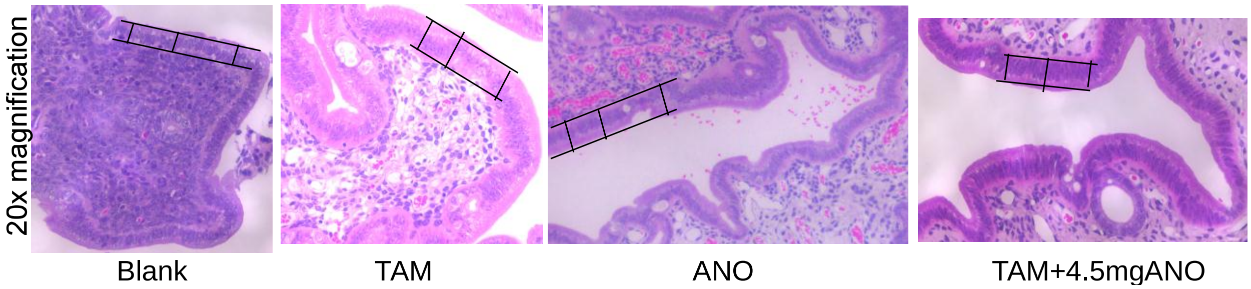

SI1 fig. 1. The average of EEC height at three marked sites of each section was used for statistical analysis as the same experiments in Fig.1 a&d. The pictures were taken at low magnification times from Paraffin-embedded H&E sections of mice uteri.

SI1 fig. 2

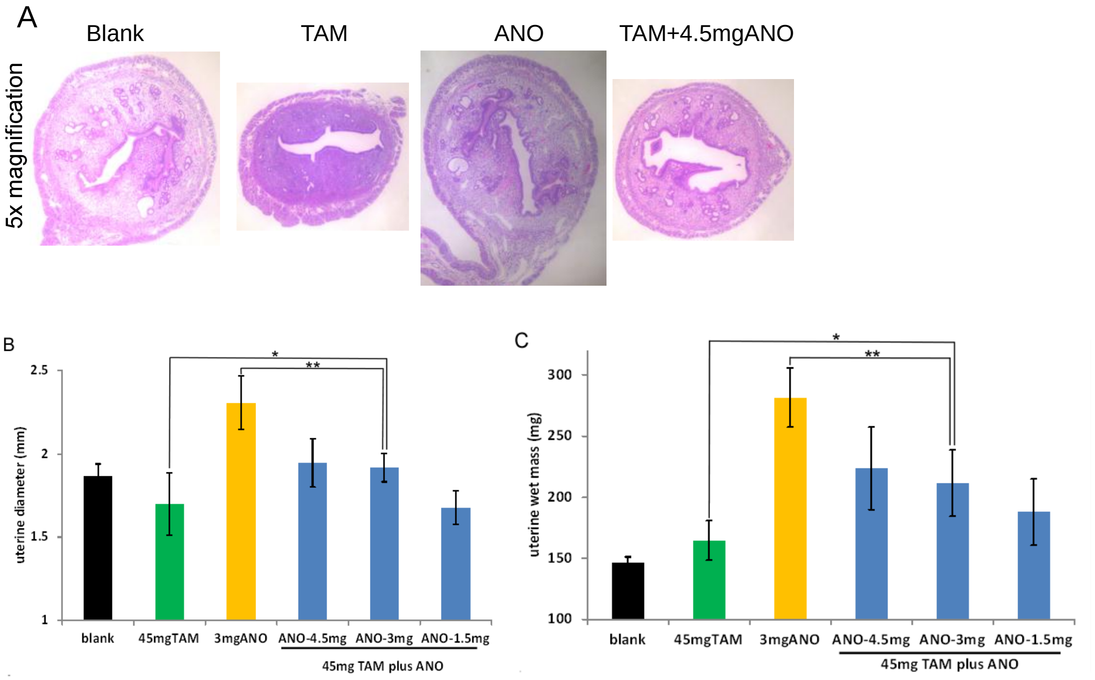

SI1 fig. 2. Tamoxifen inhibited anordrin-induced uterine hypertrophy in normal mice. (A) The images of the Paraffin-embedded H&E sections of mice uterus. (B) Statistical analysis of uterus

diameter (mm) of The Paraffin-embedded H&E sections as (A). N=2x6. * and ** mean P<0.05 and P<0.01, respectively. (C) Statistical analysis of uterus wet mass (mg). N=2x6. * and ** mean P<0.05 and P<0.01, respectively.

SI1 fig. 3

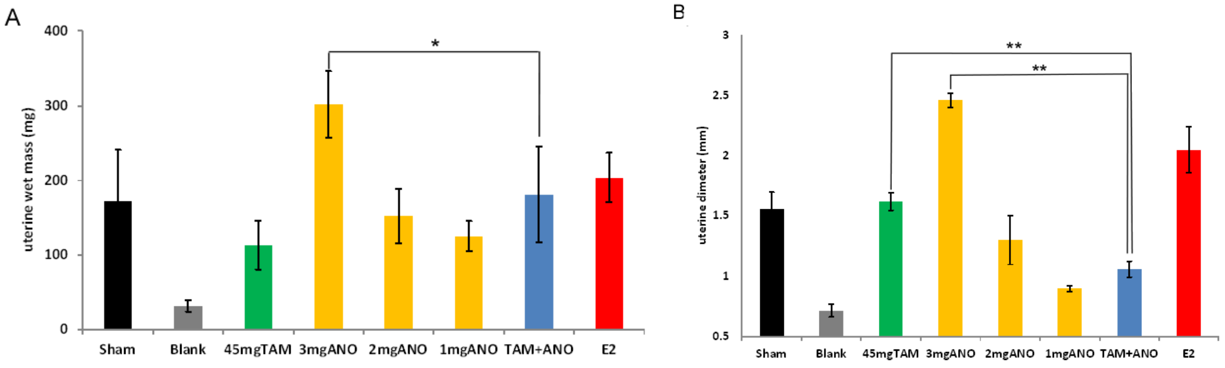

SI1 fig. 3. Tamoxifen inhibited anordrin prevention of uterine atrophy in OVX mice. Statistical analysis of uterus wet mass (A) and diameter (B) of The Paraffin-embedded H&E sections was showed from the same experimental groups as Fig.1 d&e. N=2x6. * and ** mean P<0.05 and P<0.01, respectively.

SI1 fig. 4

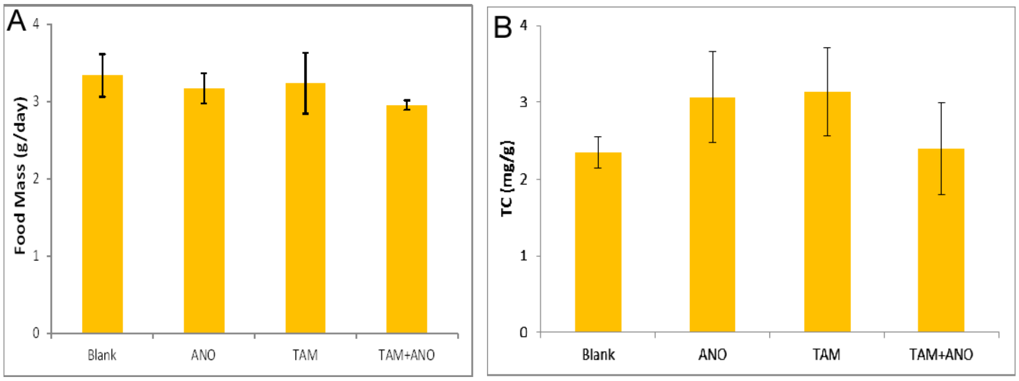

SI1.fig. 4. Statistical analysis of (A) daily food intake per mice and (B) total cholesterol (TC) indicated as in Figure 2.N=2x6.

SI1 fig. 5

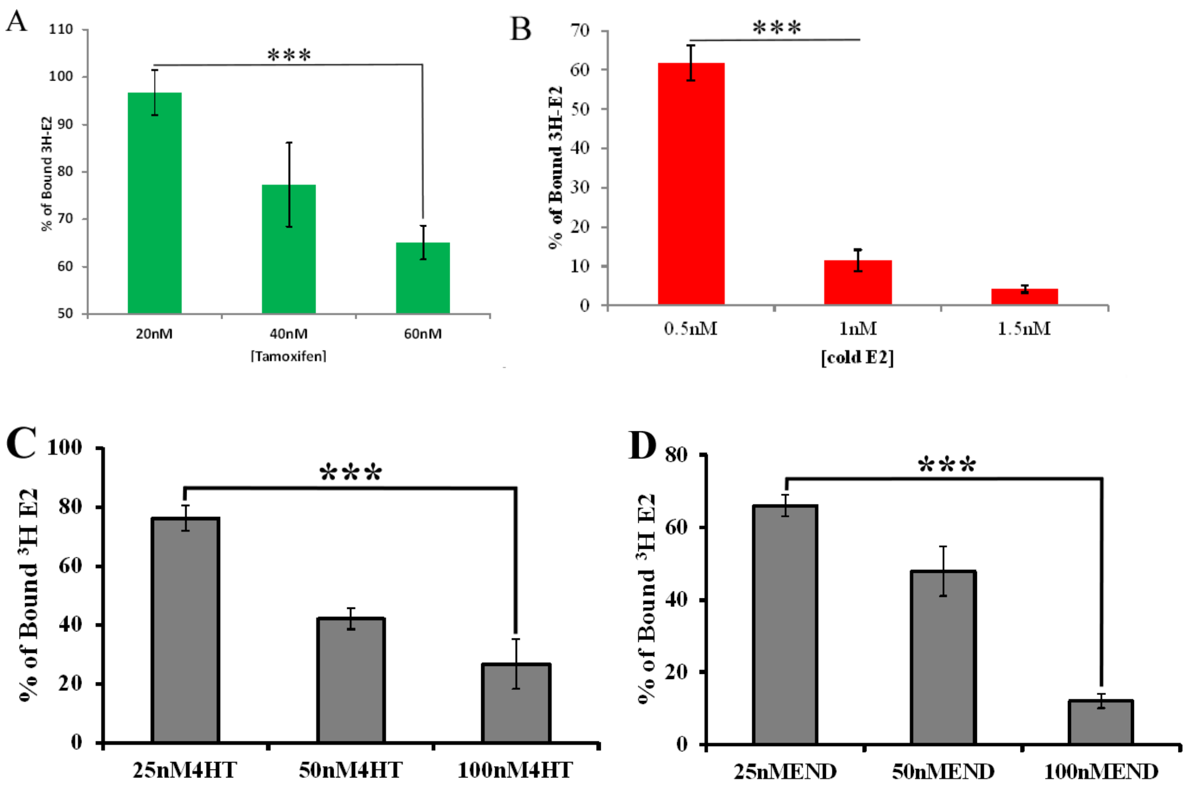

SI1.fig. 5. E2, tamoxifen and tamoxifen metabolites bound to ER-α-36. (A) The percent of ^{3}H-E2 binds to ER-α-36 competed by tamoxifen (TAM) to be normalized with ^{3}H-E2 only (blank) after subtracting the DPM of ^{3}H-E2 from equal amount of total cellular protein. N=3x3. *** means P<0.001. (B) The percent of ^{3}H-E2 binds to ER-α-36 competed by cold E2 to be normalized with ^{3}H-E2 only (blank) after subtracting the DPM of ^{3}H-E2 from equal amount of total cellular protein. N=3x3. *** means P<0.001. (C) The percent of ^{3}H-E2 binds to ER-α-36 competed by 4-hydroxytamoxifen (4HT) to be normalized with ^{3}H-E2 only (blank) after subtracting the DPM of ^{3}H-E2 from equal amount of total cellular protein. N=2x3. *** means P<0.001. (D) The percent of ^{3}H-E2 binds to ER-α-36 competed by endoxifen (END) to be normalized with ^{3}H-E2 only (blank) after subtracting the DPM of ^{3}H-E2 from equal amount of total cellular protein. N=2x3. *** means P<0.001.

SI2. Dinordiol ($_d$IV) and anordrin (compound aV) was synthesized using the following steps:

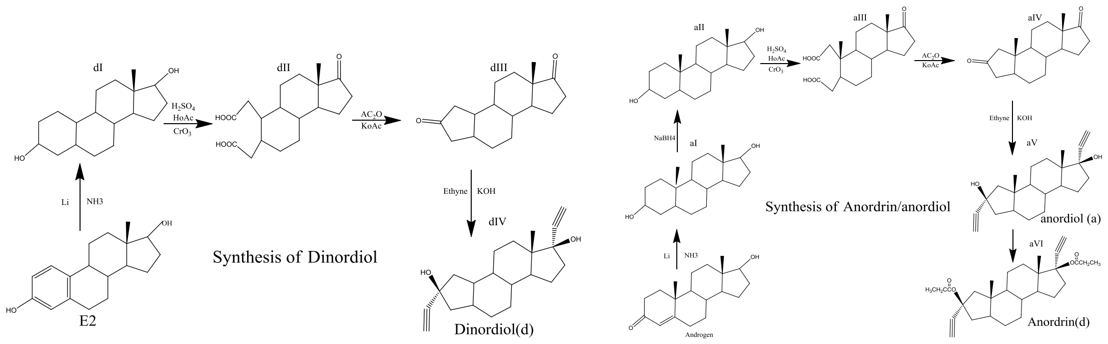

Supplement: Supplementary Information [file srep43940-s1.pdf]
